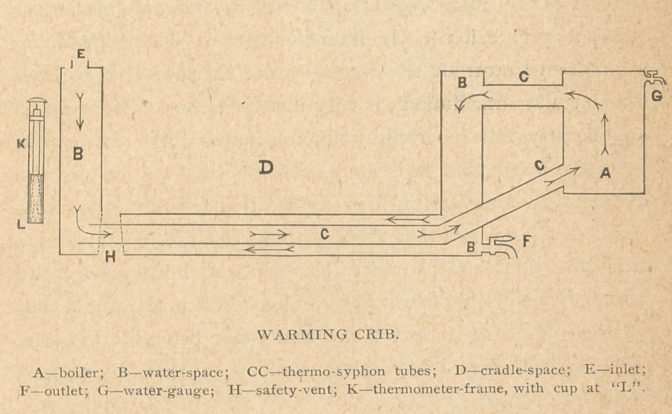# The Warming-Crib

**Published:** 1887-05

**Authors:** John Bartlett

**Affiliations:** Professor of Diseases of Women and Children at the Chicago Policlinic; 482 North Clark Street


THE CHICAGO

MEDICAL JOURNAL AND EXAMINER.

VOL. LIV. MAY, 1887. No. 5

### ORIGINAL COMMUNICATIONS.

### THE WARMING-CRIB.

BY JOHN BARTLETT, M. D., PROFESSOR OF DISEASES OF WOMEN AND CHILDREN AT THE CHICAGO POLICLINIC.

[Read before the Chicago Medical Society, May 2nd, 1887.]

While probably for ages it has been the custom on the part of the attendants of women in confinement to secure additional warmth to immature or feeble children, the plan of placing them in a receptacle convenient for maintaining a constant elevated temperature is not common. Such an arrangement, we are told, has long been used at Moscow. Denucé described an incubator in 1857; Credé has used one for a score of years; and, of late, the somewhat complicated incubator of Tarnier has come prominently into notice.

Recently, having urgent occasion to use one of these warming cribs I was called upon to give directions for the manufacture of it. In the literature accessible I found no details sufficient to guide the mechanic, and I was forced to guess at the dimensions of the various parts of the apparatus. With this experience of the need of proper directions for the making of a suitable cradle, I have deemed it worth while to exhibit one of these useful appliances, and, what is of more importance, to furnish a diagram of one, with measurements, from which an apparatus that will answer all expectations may be constructed by any intelligent artisan.

Credé's cradle consists of two small bathing-tubs placed one within the other, with a water-space between them. It is used by filling the interspace with water at 122 degrees. In four hours this water is withdrawn and the space again filled with a new supply, or the cooling water can be withdrawn by degrees and hot water added as needed. In Tarnier's apparatus, the child, in a cradle-frame, is enclosed in a duly ventilated case made partly of glass ; beneath this is a tank of water provided with a boiler and thermo-syphon, kept warm by means of a lamp. It is necessary to keep the lamp lighted for a number of hours, and then to discontinue its use for a time, lest the incubator become too hot. Tarnier has modified the apparatus for use in private practice by dispensing with the lamp and replacing the large hot-water tank with a number of smaller flasks. It is necessary to empty and refill one of these every half hour in order to maintain the proper temperature in the cradle-space. It will be seen that each of these three varieties requires a good deal of watchfulness and care.

The crude warming-crib which I present this evening is simple, efficient, safe and, perfectly easy of management. It consists, as you see, of an outer case of galvanized iron, or preferably of copper, and an inner smaller one. Between the two cases is the water area, closed in all around. Standing off some inches from the outer box will be noticed a small boiler connected by tubes with the water-space. The upper of these tubes terminates immediately after entering the water-area. The lower one extends through the lower portion of the water-space, to end near the opposite side of the box. This arrangement is necessary in order to form an effective thermo-syphon by means of which circulation of the water in the water-space may be maintained, and thus an equable temperature of all parts of the crib secured.

WARMING CRIB.

A—boiler; B—water-space; CC—thermo-syphon tubes; D—cradle-space; E—inlet; F—outlet; G—water-gauge; H—safety-vent; K—thermometer-frame, with cup at "L".

MEASUREMENTS FOR SMALLER CRIB RECOMMENDED.

Outside measurement of crib (the figures have reference to the metal case), 26x26 inches; outside depth, 11½ inches; size of cradle-space, 21x21 inches; depth of cradle-space, 9 inches; width of water-space, 2½ inches; boiler, 5 inches in diameter, and 7½ inches long. Pipes, 1⅛ inches in diameter. Safety-vent, 1⅛ inches at top; at bottom, 1⅝ inches. Capacity of water space, 12 gallons. Distance of the boiler from the crib wall, 5 inches.

MEASUREMENTS OF THE LARGER CRIB EXHIBITED.

Outside measurement, 30x32 inches; outside depth, 14 inches; size of cradle-space, 22x24 inches; depth of cradle-space, 10 inches; width of water-space, 4 inches. Boiler, 5 inches in diameter, and 9½ inches long. Pipes, 1¼ inches in diameter. Safety-vent, 1¼ inches at the top; at bottom 1⅞ inches. Capacity of water-space, 30 gallons. Distance of the boiler from the crib wall, 5 inches.

NOTE.—In arranging the bottom board and castors the carpenter should see to it, that the boiler stands at the proper height to accommodate the lamp to be used. The "Florence Lamp" renders good service.

Leakage into the cradle-space, occurring unobserved, might prove fatal to the occupant. To anticipate such an accident a safety vent is provided. A conical tube is set, with the larger orifice downward, in the bottom of the crib-space. To prevent the ingress of cold air it is packed lightly with absorbent cotton. In the event of water finding its way into the cradle-space it would run into the vent, wet the cotton, and cause it to fall out of the tube, leaving this free to act as a drain.

One of the necessary attachments to such an apparatus is what may be called a thermometer frame. The determining of the temperature of the water by the thermometer, without this peculiar attachment, is very unsatisfactory. The column of mercury falls so rapidly upon removing the instrument from the water that it is impossible in this way to read the temperature accurately. To obviate this difficulty, the expedient has been resorted to of placing the thermometer in a frame-work of metal, so that the bulb and lower part of the stem of the instrument rest in a cup. When the frame and thermometer are removed, the hot water brought up in the cup causes the mercury to retain its level, so that this may be noted at leisure.

It is desirable to encase the metal box with wood, both that the heat may be better retained, and that additional strength be secured. The metal walls are not strong enough properly to sustain the pressure of a column of water ten inches high. They bulge in all directions, indicating considerable strain at their seams. The upward pressure on the bottom of the cradle-space in this incubator is 200 pounds. The outer bottom and sides are therefore sustained by boards, and pressure upward upon the cradle-space bottom is opposed by wooden standards, resting upon the ends of cross strips fitted to this bottom and held down by slats crossing from side to side over the top of the box.

To use the apparatus, the water-space is filled with warm water, preferably by means of rubber tubing, and the lamp lighted. The water is never changed, and no attention is required further than to so adjust the wick of the lamp as to maintain the bed of the infant at the temperature desired. With a little practice the flame of the lamp can be so adjusted by the eye, and the temperature of the incubator-wall so estimated by the hand, that it will hardly be found necessary to consult the thermometer. A given temperature of the infant's bed may be maintained within a few degrees with very little trouble. The volume of water is so large that variations of temperature of the room, or in the amount of heat applied, are slow to induce a change in the temperature of the bed. In this one a temperature of 92 degrees was generally maintained. It varied between 92 and 86 degrees. It was gradually allowed to fall to the latter point during a number of days preceding the final removal of the infant from the cradle. A temperature of 122 degrees in the water indicated 92 degrees in the bed; the air of the room being at 70 degrees. The gas stove or lamp used under the boiler should be capable of furnishing an abundance of heat; for the large crib here shown a coal-oil lamp with a four-inch wick was amply sufficient. It is convenient to have two of these lamps, so that one may be always ready when needed. The bed is probably best made of horse-hair or cotton; it fills up about one-half of the cradle-space. The babe, dressed as usual, is covered by a woolen cloth, and over this rests a blanket sufficiently large to cover the whole top of the incubator, and thus to roof in the cradle-space, except about the face of the infant.

The incubator may be emptied of water by means of tubing attached to the discharge-cock. The crib here presented has disadvantages. Intended for triplets, it is very capacious. Its water-space, with a capacity of 30 gallons, is probably unnecessarily large. This renders it difficult to be filled and emptied, and cumbersome to be moved. Probably one of considerably smaller dimensions would be preferable. To the wood-cut prepared to accompany this paper, as published, are appended dimensions for a crib of, as is supposed, a more desirable size.

482 North Clark Street.

## Figures and Tables

**Figure f1:**